# Formaldehyde Stress Responses in Bacterial Pathogens

**DOI:** 10.3389/fmicb.2016.00257

**Published:** 2016-03-03

**Authors:** Nathan H. Chen, Karrera Y. Djoko, Frédéric J. Veyrier, Alastair G. McEwan

**Affiliations:** ^1^School of Chemistry and Molecular Biosciences, The University of Queensland, St LuciaQLD, Australia; ^2^INRS-Institut Armand-Frappier, Institut National de la Recherche Scientifique, Université du Québec, LavalQC, Canada

**Keywords:** formaldehyde, glutathione, host–pathogen interactions, *Neisseria*, *Haemophilus*

## Abstract

Formaldehyde is the simplest of all aldehydes and is highly cytotoxic. Its use and associated dangers from environmental exposure have been well documented. Detoxification systems for formaldehyde are found throughout the biological world and they are especially important in methylotrophic bacteria, which generate this compound as part of their metabolism of methanol. Formaldehyde metabolizing systems can be divided into those dependent upon pterin cofactors, sugar phosphates and those dependent upon glutathione. The more prevalent thiol-dependent formaldehyde detoxification system is found in many bacterial pathogens, almost all of which do not metabolize methane or methanol. This review describes the endogenous and exogenous sources of formaldehyde, its toxic effects and mechanisms of detoxification. The methods of formaldehyde sensing are also described with a focus on the formaldehyde responsive transcription factors HxlR, FrmR, and NmlR. Finally, the physiological relevance of detoxification systems for formaldehyde in bacterial pathogens is discussed.

## Introduction

Formaldehyde (H_2_C = O), structurally the simplest of all aldehydes, is a major byproduct of the manufacturing industry (Heck et al., 1990), a common environmental hazard (Flyvholm and Andersen, 1993; Tang et al., 2009; International Agency for Research on Cancer, 2012), and a product of the cellular metabolism of many methylated compounds (see Potential Sources of Formaldehyde). In the scientific literature, studies relating to formaldehyde have focused almost exclusively on its toxicology in animals and humans. The carcinogenic properties and detrimental effects of formaldehyde exposure on growth and reproductive development have been described and summarized extensively (Golden et al., 2006; Tang et al., 2009; Zhang et al., 2009; Szende and Tyihak, 2010; Duong et al., 2011; Tulpule and Dringen, 2013). Formaldehyde is also highly toxic to microbes and it has widespread application as a disinfectant for sterilization. Of interest in this review are the adaptive responses to formaldehyde that occur in microbes, especially bacterial pathogens.

Although it is often considered in a toxicological context, formaldehyde is an important cellular metabolite. In the bacterial world, formaldehyde is generated by methanotrophs and methylotrophs during the oxidation of short-chain hydrocarbons such as methane or methanol. Thus, details of the metabolic reactions and physiological fate of this aldehyde are available mostly in the context of methane or methanol catabolism (Vorholt et al., 2000; Marx et al., 2003, 2004). However, recent discovery of inducible formaldehyde detoxification systems in bacteria that do not use methane or methanol as a carbon source highlights the significance of this aldehyde in the general physiology of prokaryotes. This review will summarize contemporary findings in this area and assess the potential role of formaldehyde during interactions between bacterial pathogens and their host.

## Mechanisms of Formaldehyde Toxicity

**FIGURE 1:**
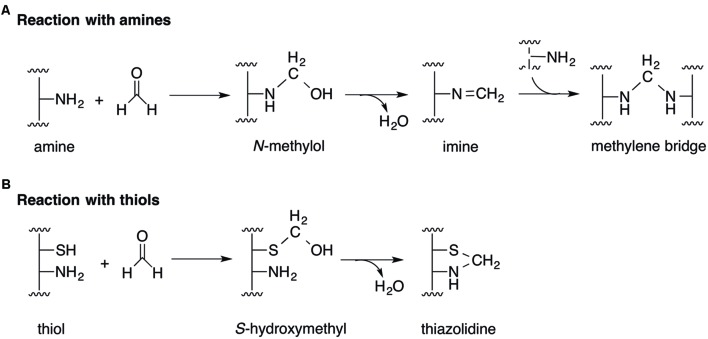
**Mechanisms of formaldehyde toxicity.** **(A)** The reaction of formaldehyde with amines forms an imine adduct via an *N*-methylol intermediate. The imine can react further with other amines to form methylene bridges between protein and DNA molecules. **(B)** The reaction between formaldehyde and thiols forms *S*-hydroxymethyl and thiazolidine adducts.

The toxicity of formaldehyde in cells arises primarily from its reactivity as an electrophile. It reacts rapidly with free thiol (-SH; Klatt and Lamas, 2000; Moran et al., 2001; Paget and Buttner, 2003) and amine (-NH_2_; Feldman, 1973; Conaway et al., 1996) groups on proteins and DNA. The nucleophilic addition of an amine to formaldehyde generates an *N*-methylol adduct that may subsequently condense to an imine (**Figure 1A**). This imine carbon is susceptible to further nucleophilic addition by a second amine, forming an irreversible cross-link composed of a methylene bridge (**Figure 1A**; Feldman, 1973; Conaway et al., 1996). In the reaction between formaldehyde and thiols, nucleophilic addition of the sulfur atom to the aldehyde forms a hemithioacetal (*S*-hydroxymethyl adduct), which may cyclize rapidly and irreversibly with a neighboring amine to generate a thiazolidine adduct (**Figure 1B**; Kallen, 1971; Higgins and Giedroc, 2014). Indeed, formaldehyde exposure has been shown to result in DNA and protein damage (Casanovaschmitz et al., 1984; Casanova et al., 1994; Yu et al., 2015), including formation of irreversible formaldehyde adducts (Heck et al., 1990) as well as formaldehyde-catalyzed DNA-DNA (Lu et al., 2010), DNA-protein (Solomon and Varshavsky, 1985; Loshon et al., 1999; Heck and Casanova, 2004), and protein-protein cross-links (Metz et al., 2004).

## Biochemical Strategies for Formaldehyde Tolerance in Bacteria

### Mechanisms of Formaldehyde Detoxification and Assimilation

**FIGURE 2:**
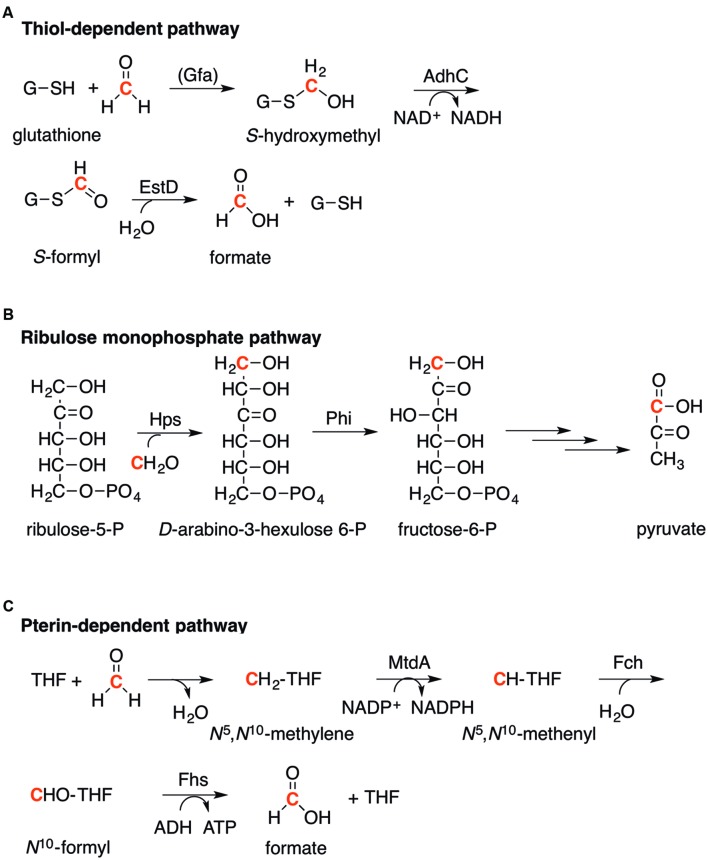
**Pathways for the detoxification of formaldehyde.** **(A).** Thiol-dependent pathway, as exemplified by glutathione (GSH). **(B).** Ribulose monophosphate (RuMP) pathway. **(C).** Pterin-dependent pathway, as exemplified by tetrahydrofolate (THF). **(A–C).** The formaldehyde carbon is highlighted in red.

Three major bacterial pathways for formaldehyde detoxification have been identified: thiol-dependent, ribulose monophosphate (RuMP)-dependent, and pterin-dependent. The stepwise transformations of formaldehyde within these pathways are well understood (**Figure 2**). Each proceeds by initial capture of formaldehyde as a less reactive derivative, which is assimilated subsequently into the usual pathways for carbon metabolism (in the case of the RuMP pathway), or is detoxified to formate (in the pterin- and thiol-dependent pathways).

#### Thiol-Dependent

The most widespread and perhaps the best characterized pathway for formaldehyde detoxification employs reactive thiols as the initial formaldehyde acceptor. In most microorganisms, this thiol is the tripeptide glutathione (GSH, *L-*γ-glutamyl-*L*-cysteinylglycine). The first step is the nucleophilic addition of GSH to formaldehyde to form *S*-hydroxymethylglutathione (HMGS; **Figure 2A**; Mason et al., 1986). This reaction occurs spontaneously but in certain bacterial species, such as *Paracoccus denitrificans* and *Rhodobacter sphaeroides*, it is catalyzed by a formaldehyde-activating enzyme (Gfa, EC 4.4.1.22; Goenrich et al., 2002; Wilson et al., 2008). The HMGS adduct is oxidized subsequently by a zinc-containing, NAD^+^-dependent alcohol dehydrogenase (AdhC, EC 1.1.1.284) to generate the thioester *S-*formylglutathione (SFG; Uotila and Koivusal, 1974a). Formate is produced and GSH is regenerated finally upon hydrolysis of SFG (**Figure 2A**). This final step is catalyzed by an esterase or *S*-formylglutathione hydrolase (EstD, EC 3.1.2.12; Uotila and Koivusal, 1974b).

GSH-dependent systems for formaldehyde detoxification were first thought to exist exclusively in environmental bacteria such as Gram-negative methylotrophs but they have now been identified in a diverse range of microorganisms, many of which do not oxidize methanol (Uotila and Koivusal, 1974a; Kaulfers and Marquardt, 1991; Fernandez et al., 1993; Gutheil et al., 1997). These include the FrmAB system in *Escherichia coli* (Herring and Blattner, 2004; Gonzalez et al., 2006), the NmlR regulons in the human pathogens *Haemophilus influenzae* and *Neisseria meningitidis* (Kidd et al., 2012; Chen et al., 2013), and the AdhR regulon in *Bacillus subtilis* (Huyen et al., 2009), each of which is described further in section “Organization and Functional Regulation of Genes in the Glutathione-Dependent Pathways.”

In bacteria that do not use glutathione, the alternative thiol mycothiol (MSH) or bacillithiol (BSH) is used as the formaldehyde carrier (Sakuda et al., 1994; Newton et al., 2009). MSH and BSH contain glycoside linkages between *N*-acetylated cysteine, D-glucosamine, and myo-inositol moieties (MSH), or between *L*-cysteine, D-glucosamine, and malic acid (BSH). A MSH-dependent homolog of AdhC has been described in *Mycobacterium smegmatis* (AdhE2, EC 1.2.1.66) and in the actinomycete *Corynebacterium glutamicum* (FadH; Norin et al., 1997; Vogt et al., 2003; Lessmeier et al., 2013). However, an *S*-formylmycothione hydrolase has not been identified in these organisms. Nevertheless, formate and MSH have been detected as the final products of formaldehyde oxidation in *M. smegmatis*, presumably as a consequence of the spontaneous degradation of *S*-formylmycothione (Vogt et al., 2003). Similarly, a BSH-dependent homolog of AdhC has been identified in *B. subtilis* (AdhA, EC 1.1.1.-) (Huyen et al., 2009) but the corresponding *S*-formylbacillithione hydrolase has not been identified. Whether formate is generated as the final oxidation product is yet to be determined.

#### RuMP Pathway

The RuMP pathway comprises two enzymes, 3-hexulose-6-phosphate synthase (Hps, EC 4.1.2.43) and 6-phospho-3-hexuloisomerase (Phi, EC 5.3.1.27) [**Figure 2B**; reviewed in (Kato et al., 2006)]. This pathway was first described in methylotrophic bacteria and archaea that use formaldehyde as a sole carbon source but it has now been identified in non-methylotrophs such as *B. subtilis* [annotated as HxlA (Hps) and HxlB (Phi)] and *Burkholderia cepacia* (Mitsui et al., 2003; Yurimoto et al., 2005; Huyen et al., 2009). In this pathway, formaldehyde is captured initially by Hps-catalyzed condensation with the C1 carbon of ribulose-5-phosphate to form *D*-arabino-3-hexulose-6-phosphate. This product is isomerized by Phi to generate fructose-6-phosphate, which is shuttled subsequently into the bacterium’s glycolytic pathways. The initial formaldehyde acceptor ribulose-5-phosphate is regenerated from fructose-6-phosphate and glyceraldehyde-3-phosphate via a series of transketolase, transaldolase, and isomerization reactions (Kato et al., 2006). The use of sugar phosphates in formaldehyde detoxification has also been identified in eukaryotic microbes such as *Candida* sp. However, xylylose-5-phosphate is used as the initial formaldehyde acceptor (Veenhuis et al., 1983).

#### Pterin-Dependent

The pterin-dependent pathway for formaldehyde detoxification takes advantage of the reactivity of formaldehyde with amines, such as that present in the pterin moiety of tetrahydrofolate (THF; **Figure 2C**). The spontaneous condensation between formaldehyde and a secondary amine in the pterin forms *N*^5^,*N*^10^-methylene-THF, which is in turn oxidized to *N*^5^,*N*^10^-methenyl-THF. The latter is catalyzed by a dehydrogenase (MtdA, EC 1.5.1.5) using NADP^+^ as the electron acceptor. *N*^5^,*N*^10^-methenyl-THF is hydrolyzed further to *N*^10^-formyl-THF by a cyclohydrolase (Fch, EC 3.5.4.9). In the final step, *N*^10^-formyl-THF is hydrolyzed by formate THF ligase (or formyl THF synthetase, Fhs, EC 6.3.4.3) to generate formate as the final product and regenerate THF (**Figure 2C**; Vorholt, 2002).

The three enzymes depicted in **Figure 2C** – MtdA, Fch, and Fhs – constitute the central pathway for methyl transfer, which is required for the synthesis of purines and amino acids, and for the initiation of protein translation. As such, this pathway is widely distributed in the bacterial world. In species that already possess the thiol- or RuMP-linked pathways, the THF-dependent pathway for methyl transfer may still act as a secondary or auxiliary system for the removal of formaldehyde. In methanotrophs and methylotrophs such as *Methylobacterium* sp. and *Hyphomicrobium* sp., this THF-linked pathway is upregulated in the presence of methane or methanol, presumably to cope with the production of formaldehyde during methane or methanol oxidation (Vorholt, 2002). Certain methanogenic archaea and methylotrophic proteobacteria use tetrahydromethanopterin (THMP) in place of THF (Maden, 2000; Vorholt, 2002). The two pterins are structurally related and molecular details of the THF- and THMP-linked pathways are analogous.

#### Direct Oxidation of Formaldehyde to Formate

In several bacterial species such as *Pseudomonas putida*, *P. aeruginosa*, and *Burkholderia fungorum*, the oxidation of formaldehyde to formate occurs in a single step that is independent of thiol, pterin, or RuMP (Ando et al., 1979; Marx et al., 2004; Liao et al., 2013). This process is catalyzed by a zinc-dependent formaldehyde dehydrogenase (EC 1.2.1.46) using NAD^+^ as the electron acceptor (Liao et al., 2013).

### Mechanisms of Formaldehyde Sensing

The various pathways for formaldehyde detoxification operate under the control of formaldehyde-responsive transcriptional factors but the biochemical mechanisms for formaldehyde sensing remain poorly understood. Transcriptional response to formaldehyde relies typically on the presence of one or more conserved cysteine thiols. Mutation of this cysteine leads invariably to a failure to respond to exogenous formaldehyde or formaldehyde generators, but how this cysteine detects formaldehyde remains unknown. Based on current understanding of other families of cysteine-based transcriptional sensors, it has been speculated that this conserved cysteine may be *S-*alkylated. The mechanism for *S*-alkylation of cysteine by formaldehyde would be analogous to that described earlier for the reaction between formaldehyde and glutathione (see **Figure 1B**). Alternatively, this cysteine may also be *S*-alkylated by a downstream product of formaldehyde such as HMGS or SFG (see **Figure 2A**) or by a product of the toxic reactions between formaldehyde and a cellular target. To date, there has been no evidence of any such *S-*modification *in vitro* or *in vivo*. The available knowledge of formaldehyde sensing in bacteria is outlined below.

#### HxlR

HxlR from *B. subtilis* controls the expression of *hxlAB*, which encodes for the RuMP pathway for formaldehyde assimilation (Yurimoto et al., 2005). It is a member of the MarR/DUF24 family of repressors that sense reactive oxygen (ROS) and electrophilic species (RES; Antelmann and Helmann, 2011; Hillion and Antelmann, 2015), as exemplified by OhrR from *B. subtilis* (Fuangthong and Helmann, 2002) and *Xanthomonas campestris* (Panmanee et al., 2006). *In vitro*, exposure of OhrR to ROS such as organic peroxides was shown to result in the oxidation of the conserved cysteine (Cys15) to a sulfenic acid (-SOH; Fuangthong and Helmann, 2002). This sulfenic acid reacts further with a second thiol, either from BSH or from a cysteine on a neighboring OhrR monomer, to form a disulfide (-S-S-), which in turn leads to dissociation of OhrR from DNA and thus derepression of gene expression (Panmanee et al., 2006; Lee et al., 2007; Newberry et al., 2007). Although HxlR also contains a conserved cysteine (Cys11) near the *N*-terminus, the reaction between formaldehyde and a cysteine thiol is not likely to generate a sulfenic acid intermediate (cf. see Mechanisms of Formaldehyde Toxicity and **Figure 1B**).

#### FrmR

**FIGURE 3:**
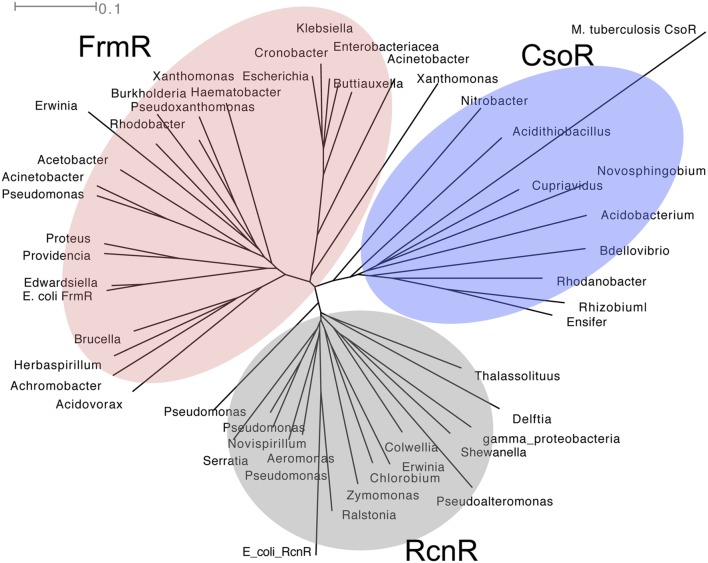
**Phylogenetic tree of CsoR (shaded in blue), RcnR (gray), and FrmR (red) family of regulators.** Amino acid sequences were aligned using ClustalX 2.1 (Larkin et al., 2007) and analyzed using SplitsTree4 (Huson and Bryant, 2006). The tree shown was drawn using the ConsensusTree function and 500 bootstrap cycles.

FrmR regulates the expression of *frmAB*, the GSH-dependent pathway for formaldehyde detoxification in *E. coli* (Herring and Blattner, 2004). Its homologs, along with the complete FrmAB pathway, have also been identified in pathogens such as *P. aeruginosa* and *Klebsiella pneumoniae* (**Figure 3**). FrmR is a member of the CsoR/RcnR family of metal ion-sensing transcriptional repressors (**Figure 3**). Prototypes of this family possess a conserved cysteine within X-Cys-His-Cys or His-Cys-His-His motifs for binding the cognate metal ion (Liu et al., 2007; Iwig et al., 2008). The conserved cysteine in FrmR from *Salmonella enterica* sv. Typhimurium (Cys35) was found to bind Co(II) and Zn(II) *in vitro* [*K*_Co(II)_ = 7.6 × 10^-6^ M; *K*_Zn(II)_ = 1.7 × 10^-10^ M] but this protein was unable to compete with dedicated metal sensors such as RcnR [*K*_Co(II)_ = 5.1 × 10^-10^ M] and ZntR [*K*_Zn(II)_ = 3.2 × 10^-12^ M; Osman et al., 2015). To date, the relevance of metal ion binding to formaldehyde sensing by FrmR remains undefined. Instead, it has been hypothesized that Cys35 reacts with formaldehyde directly to form an *S*-hydroxymethyl adduct and, in the presence of a neighboring primary amine, a thiazolidine-like adduct (see **Figure 1B**; Higgins and Giedroc, 2014). Only one CsoR/RcnR homolog has been demonstrated to detect non-metals using Cys35 (Luebke et al., 2014). This is CstR, a persulfide sensor that controls sulfide homeostasis in *Staphylococcus aureus* (Luebke et al., 2014).

#### NmlR

**FIGURE 4:**
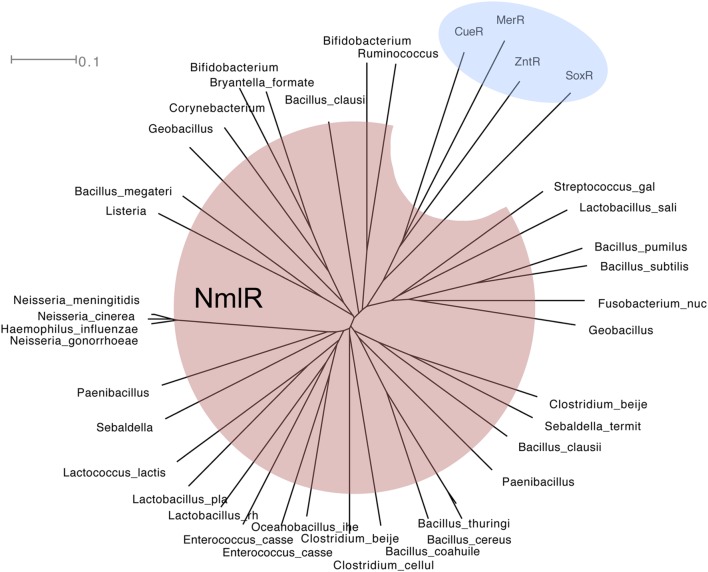
**Phylogenetic tree of MerR (shaded in blue) and NmlR (red) family of regulators.** Amino acid sequences were aligned using ClustalX 2.1 (Larkin et al., 2007) and analyzed using SplitsTree4 (Huson and Bryant, 2006). The tree shown was drawn using the ConsensusTree function and 500 bootstrap cycles.

NmlR controls the expression of the GSH-dependent pathway for formaldehyde detoxification. It was first identified in pathogenic *Neisseria* species but its homologs have now been found in several medically significant human pathogens, including *H. influenzae*, *Streptococcus pneumoniae*, *Lactobacillus* sp., and *Clostridium* sp. (Kidd et al., 2005, 2012; Stroeher et al., 2007; McEwan et al., 2011; Chen et al., 2013). NmlR homologs form a clade within the diverse family of MerR repressor-activators that respond to a wide range of molecules, including soft transition metal ions, the superoxide anion, and drug-like compounds (**Figure 4**; Ahmed et al., 1994, 1995; Hidalgo and Demple, 1994; Brown et al., 2003; McEwan et al., 2011). Members of the NmlR clade are thought to sense oxidative and/or carbonyl stressors (Kidd et al., 2005; Stroeher et al., 2007; Huyen et al., 2009).

As is the norm for all known formaldehyde sensors, there is absolute conservation of a cysteine within the NmlR clade [Cys54 in NmlR from *H. influenzae* or Cys52 in NmlR (AdhR) from *B. subtilis*]. Mutation of Cys54 to an Ala in the homolog from *H. influenzae* led to an enhanced sensitivity to growth inhibition and a failure to activate the expression of AdhC in the presence of formaldehyde (manuscript submitted). Likewise, a mutant strain of *B. subtilis* carrying the C52A variant of AdhR was unable to generate an *adhA* (*adhC*) transcript in response to challenge with formaldehyde (Huyen et al., 2009). For both NmlR and AdhR, no evidence of *S*-alkylation by formaldehyde, formaldehyde generators, or downstream formaldehyde detoxification products has been reported thus far.

## Genetic and Functional Basis for Formaldehyde Detoxification via Glutathione-Dependent Pathways

Amongst the three pathways for the detoxification of formaldehyde, the GSH-dependent pathway is the most widely distributed in the biological world, with examples from bacteria, plants, and mammals (Uotila and Koivusal, 1974a; Harms et al., 1996; Gutheil et al., 1997; Barber and Donohue, 1998b; Cummins et al., 2006; Chen et al., 2013). As outlined briefly in section “Mechanisms of Formaldehyde Detoxification and Assimilation,” three separate enzymes catalyze the consecutive steps of the oxidation of formaldehyde to formate. These are the formaldehyde-activating enzyme Gfa, the alcohol dehydrogenase AdhC, and the thioesterase EstD (**Figure 2A**). The biochemical properties of each of these enzymes have been fairly well characterized and are summarized in this section. Although this core pathway is conserved, the organization and regulation of the encoding genes are varied, and are reviewed below.

### Enzymes of the GSH-Dependent Pathway

#### Formaldehyde-Activating Enzyme (Gfa, EC 4.4.1.22)

Gfa is a zinc-dependent enzyme that accelerates the spontaneous the condensation of GSH with formaldehyde to form HMGS. It was first described in *P. denitrificans* but it has also been identified in *Sinorhizobium meliloti* and *R. sphaeroides* (Goenrich et al., 2002; Neculai et al., 2005; Wilson et al., 2008). The pseudo first-order rate constant for the formation of HMGS as catalyzed by Gfa has been estimated to be 10-fold higher than that for the spontaneous formation of HMGS (Goenrich et al., 2002). However, a recent study has suggested that this enzyme does not catalyze the formation of HMGS, but instead it may act as a GSH carrier to promote co-localization with formaldehyde within the cell (Hopkinson et al., 2015). Nevertheless, Gfa is notably absent from the GSH-dependent pathway for formaldehyde tolerance in non-methanotrophs such as pathogenic *Neisseria* and *H. influenzae*. Thus it is likely that the rate of spontaneous condensation with GSH is sufficient for the initial capture of formaldehyde in these organisms.

#### Alcohol Dehydrogenase (AdhC, EC 1.1.284)

The class III, zinc-dependent enzyme AdhC catalyzes the oxidation of HMGS to *S*-formylglutathione using NAD^+^ as the electron acceptor (**Figure 2A**). The human AdhC homolog ADH3 is particularly well characterized. ADH3 displays a wide range of specific activity in the presence of HMGS as a substrate (*k*_cat_/*K*_m_ values between 50 and 1000 μM^-1^ min^-1^; Hedberg et al., 2003; Hoog et al., 2006; Sanghani et al., 2006; Staab et al., 2008a). Intriguingly, recent biochemical studies of AdhC homologs from human, *Saccharomyces cerevisiae* and *E. coli* demonstrated that AdhC may participate in the defense against nitrosative (nitric oxide) stress, as it also catalyzes the reduction of *S*-nitrosoglutathione (GS-NO) to generate glutathione sulfinamide (GS-ONH_2_) using NADH as the electron donor (Jensen et al., 1998; Liu et al., 2001; Hedberg et al., 2003). While the relevance to formaldehyde detoxification is unclear, it has been proposed that AdhC may function as a GS-NO reductase by NAD^+^/NADH cofactor recycling by using the HMGSH oxidase pathway to regenerate NADH (Staab et al., 2008b, 2009).

#### *S*-formylglutathione Hydrolase (EstD, EC 3.1.2.12)

EstD is a Ser-His-Asp esterase. Homologs from human (ESD), *Arabidopsis thaliana* (AtSFGH), *E. coli* (FrmB), and *N. meningitidis* (EstD) hydrolyze a range of synthetic esters, including *p*-nitrophenyl acetate, 4-methylumbelliferyl acetate, and naphthyl acetate, but each displays a high specific activity (up to 10-fold higher) toward the predicted physiological substrate *S*-formylglutathione (*k*_cat_/*K*_m_ values between 0.015 and 2 × 10^6^ M^-1^ s^-1^; Uotila and Koivusal, 1974b; Cummins et al., 2006; Gonzalez et al., 2006; Chen et al., 2013). A second homolog of EstD, annotated as YeiG, is present in *E. coli*. Compared to FrmB, YeiG displayed a 20-fold higher specific activity for *S*-lactoylglutathione. *S*-lactoylglutathione itself is an intermediate in the pathway for the detoxification of methylglyoxal via the glyoxalase system (Gonzalez et al., 2006). Therefore, YeiG was hypothesized to participate in the removal of methylglyoxal. As methylglyoxal is a potential source of formaldehyde in cells (see Potential Sources of Formaldehyde), YeiG may also contribute indirectly to formaldehyde tolerance.

All EstD homologs possess a conserved cysteine that is situated in close proximity to the active site pocket but is not essential for enzyme activity (Cummins et al., 2006; Gonzalez et al., 2006; Chen et al., 2013). Recent biochemical studies with EstD from *N. meningitidis* and *A. thaliana* suggested that this cysteine (Cys54 in *N. meningitidis*) acts as a site of post-translational regulation of enzyme activity (Cummins et al., 2006; Chen et al., 2013). Cys54 is readily alkylated with agents such as iodoacetamide (Cummins et al., 2006; Gonzalez et al., 2006; Chen et al., 2013). This *S-*modification was thought to physically block substrate access to the catalytic site (Chen et al., 2013). Indeed, treatment with iodoacetamide abolished the activity of EstD completely (Cummins et al., 2006; Gonzalez et al., 2006; Chen et al., 2013). The physiological significance for these *in vitro* observations is yet to be established.

### Organization and Functional Regulation of Genes in the Glutathione-Dependent Pathways

#### AfdRS and RfdRS Regulons

**FIGURE 5:**
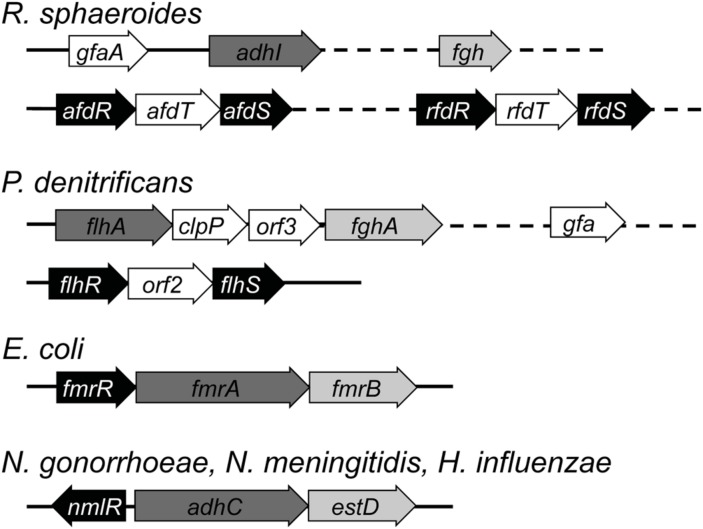
**The genetic organization of glutathione-dependent pathways for formaldehyde detoxification.** Genes encoding for the transcriptional regulators are shaded in black. Genes encoding for the glutathione-dependent alcohol dehydrogenase and the *S*-formylglutathione hydrolase are shaded in dark gray and light gray respectively. Genes that are located on different parts of the chromosome are connected using dashed lines.

The purple non-sulfur photosynthetic bacterium *R. sphaeroides* produces formaldehyde during methanol utilization. The *gfa*, *adhC* (*adhI*), and *estD* (*fgh*) genes in this bacterium are not organized in an operon (**Figure 5**). While *gfa* is adjacent to *adhI* and this arrangement is clustered with genes that encode for other metabolic enzymes such as formate dehydrogenase (Wilson et al., 2008). An *adhI* mutant strain of *R. sphaeroides* failed to oxidize formaldehyde, as demonstrated using whole-cell NMR studies in the presence of ^13^C-formaldehyde. This mutant was also unable to grow in the presence of methanol as a sole carbon source (Barber and Donohue, 1998b; Hickman et al., 2004), presumably as a consequence of the buildup of formaldehyde during methanol oxidation. The direct effect of excess formaldehyde on the growth of the *adhI* mutant is not known.

Transcriptional regulation of *gfa* and *fgh* expression in response to formaldehyde or methanol has not yet been described. In the case of *adhI*, its promoter was shown to be activated by both formaldehyde and methanol, although induction by formaldehyde occurs more rapidly and at a lower concentration (Barber and Donohue, 1998a). Expression of *adhI* is controlled by two separate, two-component regulatory systems: (i) AfdRS, which activates transcription in the presence of formaldehyde, and (ii) RfdRS, which is thought to act as a repressor, although the signal for derepression is unknown (Hickman et al., 2004). Unlike the other regulators of formaldehyde detoxification, both these systems do not seem to harbor any conserved cysteines (see Mechanisms of Formaldehyde Sensing) and thus it is unknown how they sense formaldehyde.

*afdR* and *afdS* are organized within an operon, as are *rfdR* and *rfdS*. Inserted between each pair of genes is a predicted open reading frame, *afdT* or *rfdT* (**Figure 5**), with both genes displaying high sequence identity to each other. To date, their role in the detoxification of formaldehyde is unclear, as an *rfdT* mutant of *R. sphaeroides* did not affect the expression of *adhI* in the presence of formaldehyde (Hickman et al., 2004).

#### FlhRS Regulon

The *adhC* (*flhA*) and *estD* (*fghA*) genes in *P. denitrificans* are arranged in an operon, along with two genes of unknown function, *clpP* and *orf3* (**Figure 5**; Harms et al., 1996). Expression of *flhA* and *fghA* is activated by FlhRS, a two-component regulator that displays high sequence similarity (>50%) to AfdRS and RfdRS from *R. sphaeroides* (Hickman et al., 2004). The *flhRS* operon is located away from *flhA-fghA*. Between *flhR* and *flhS* is an open reading frame, *orf2* (**Figure 5**), which shows sequence similarity to both *afdT* and *rfdT* from *R. sphaeroides*. *gfa* is also present in the genome of *P. denitrificans* but it is not part of the *flhA-fghA* operon (Goenrich et al., 2002). Whether its expression is controlled by FlhRS is yet to be defined.

A *flhRS* mutant strain of *P. denitrificans* failed to activate the expression of *flhA* and *fghA* in the presence of choline, a formaldehyde-generating substrate, as the sole carbon source (Harms et al., 2001). Inactivation of *flhRS*, *flhA*, or *fghA* each led to an inability to grow in the presence of methanol or methylamine as the sole carbon source, indicating that these genes are required for methanotrophic growth (Ras et al., 1995; Harms et al., 1996, 2001). This growth defect was not unexpected, as catabolism of methanol and methylamine both generate formaldehyde as a byproduct.

#### FrmR Regulon

The genes encoding for AdhC (*frmA*) and EstD (*frmB*) from *E. coli* are arranged in an operon, along with the gene that codes for their transcriptional regulator (*frmR*; **Figure 5**; Herring and Blattner, 2004). Exposure to formaldehyde was shown to induce robust expression of *frmAB* (over 100-fold; Herring and Blattner, 2004) and increase the activity of FrmA in whole cell extracts (Gutheil et al., 1997). Expression of *frmB* was not induced upon treatment with GSNO, hydrogen peroxide, or methyl viologen, indicating that the regulon did not respond to general oxidative or nitrosative stress (Herring and Blattner, 2004; Gonzalez et al., 2006).

#### NmlR Regulons

The *adhC-estD* operon in *N. meningitidis* (meningococcus)*, N. gonorrhoeae* (gonococcus), and *H. influenzae* is located adjacent but divergent to *nmlR*, which encodes for their transcriptional regulator (**Figure 5**; Kidd et al., 2005; Potter et al., 2007; Chen et al., 2013). Meningococcal mutant strains of *adhC* and *estD* displayed an enhanced sensitivity to growth inhibition by exogenous formaldehyde but not other aldehydes or carbonyl compounds such as methylglyoxal (Chen et al., 2013). The growth defect was more pronounced for the *estD* mutant when compared with the *adhC* single mutant or the *adhC-estD* double mutant. It was thus speculated that accumulation of *S*-formylglutathione, the substrate for EstD, is more toxic than that of HMGS, the substrate for AdhC, or than formaldehyde itself (Chen et al., 2013).

In the case of *H. influenzae*, growth of an *adhC* mutant strain was inhibited by formaldehyde, methylglyoxal, and glycolaldehyde (Kidd et al., 2012). An *nmlR* mutant strain displayed increased growth sensitivity toward formaldehyde but not methylglyoxal or glycolaldehyde when compared to the wild-type organism (manuscript submitted). AdhC activity in this pathogen is upregulated in response to both formaldehyde exposure and high oxygen tension (Gutheil et al., 1997; Kidd et al., 2012). Conversely, growth of the *adhC* mutant was suppressed by high oxygen tension in the presence of glucose as a sole carbon source but in the absence of added formaldehyde (Kidd et al., 2012). These growth conditions are known to promote the generation of dicarbonyls such as methylglyoxal (Okado-Matsumoto and Fridovich, 2000; Kidd et al., 2012), a precursor for the production of formaldehyde (see Potential Sources of Formaldehyde).

This *nmlR-adhC-estD* arrangement is not universal (McEwan et al., 2011). *estD* is absent in the human pathogen *S. pneumoniae* and there is no evidence of a formaldehyde-related phenotype in the pneumococcal *nmlR* mutant (Stroeher et al., 2007). Likewise, *estD* is not present in *B. subtilis*. Instead, NmlR in *B. subtilis*, annotated as AdhR, was found to upregulate three genes in response to methylglyoxal or formaldehyde exposure. These are *adhA, yraC*, and *yraA*, which encode for an AdhC homolog, a γ-carboxymuconolactone decarboxylase, and a cysteine proteinase, respectively (Huyen et al., 2009). YraC is proposed to be a component of protocateculate metabolism and a homolog of YraC from *Legionella pneumophila* has been shown to display peroxidase activity (Huyen et al., 2009; Chen et al., 2015). How YraC contributes to the defense against formaldehyde toxicity remains to be defined. Likewise, the role for *yraA* is not understood, although it has been hypothesized to function in the repair of formaldehyde-induced protein damage (Huyen et al., 2009).

## A Role for Formaldehyde Detoxification in Bacterial Pathogenesis

### Evidence for Horizontal Transfer of *nmlR-adhC-estD* Genes Between Pathogenic Species

The presence of inducible formaldehyde detoxification systems in non-methylotrophs, including those that cause human diseases, hints at the significance and role of this toxic aldehyde in bacterial physiology. In some pathogens, the loss or mutation of these detoxification genes led to phenotypic defects even in the absence of added formaldehyde. As already mentioned earlier, growth of the *adhC* mutant strain of *H. influenzae* was suppressed when cultured under high oxygen tension and in the presence of glucose as the sole carbon source (Kidd et al., 2012). Although a growth defect in the absence of formaldehyde was not reported for the equivalent mutant of *N. meningitidis*, the *adhC*, as well as the *nmlR* and *estD* mutant strains of this bacterium were shown to be non-viable or “aged” within mature biofilm communities (Chen et al., 2013). Together, these studies provided strong evidence, albeit indirect, that formaldehyde accumulates endogenously.

The majority of NmlR regulators identified by phylogenetic analysis are found in Gram-positive bacteria (**Figure 4**). The only examples in Gram-negative bacteria are from the *Neisseria* genus and a few *Pasteurellaceae* species. Within the *Neisseria* genus, the *nmlR-adhC-estD* locus is identified only in the lineage of meningococcal-related species (Guibourdenche et al., 1986), namely *N. meningitidis, N. gonorrhoeae*, *N. lactamica*, *N. cinerea*, and *N. polysaccharea*. Within the *Pasteurellaceae* family, the same *nmlR-adhC-estD* locus is found in *H. influenzae* and two other species, *Aggregatibacter actinomycetemcomitans*, a bacterium from the buccal normal flora that is often associated with periodontitis, and the rumen bacterium *Mannheimia succiniciproducens*. This *in silico* analysis raises the possibility that the presence of the *nmlR* operon in Gram-negative bacteria is a consequence of a gene transfer event from Gram-positive bacteria that occupy the same environmental niche.

**FIGURE 6:**
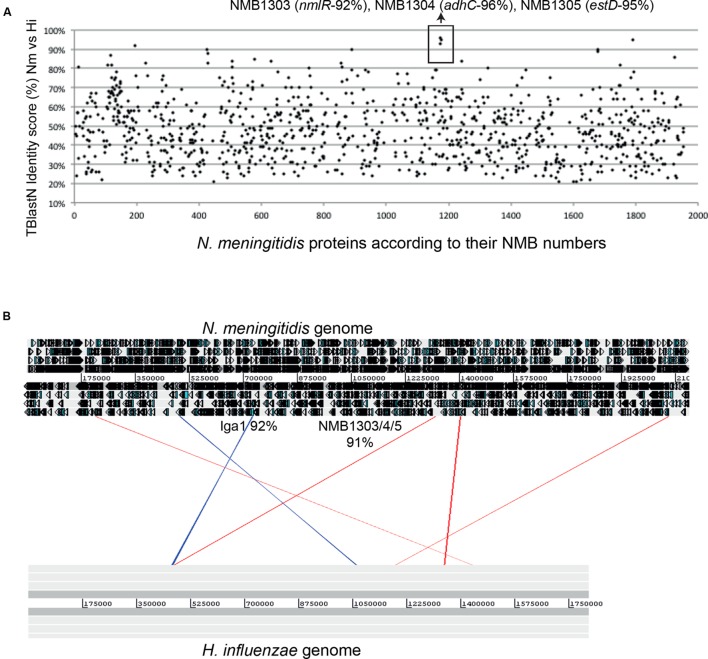
**Horizontal transfer of the *nmlR-adhC-estD* locus between *Haemophilus* and *Neisseria.*** **(A)** Graphical representation of the TBlastN identity scores of *N. meningitidis* MC58 proteins against *Haemophilus influenzae* genomes. **(B)** ACT image of BlastN genome comparison of *Neisseria meningitidis* MC58 and *H. influenzae* genomes. The strongest hits potentially representing exchange of DNA are represented.

It has been proposed that *H. influenzae* received the gene coding for an IgA protease (IgaB), a well-defined bacterial virulence determinant, by horizontal gene transfer from *N. meningitidis* (Murphy et al., 2011). It must be noted that the *nmlR-adhC-estD* locus in *H. influenzae* is found adjacent to *igaB*. Interestingly, comparison of the identity score for all proteins from *N. meningitidis* MC58 BLAST against *H. influenza*e PittEE, revealed that the NmlR, AdhC, EstD protein sequences share an abnormally high percentage of identity (**Figure 6A**). The same is true for their DNA sequences (**Figure 6B**). In addition, the surrounding regions of the *nmlR-adhC-estD* locus are conserved in other *Haemophillus* species (**Figure 6B**). Considering all the evidence presented here, these *in silico* analyses suggest recent transfer of *nmlR*, *adhC*, and *estD* from pathogenic *Neisseria* to pathogenic *Haemophilus* species.

It is notable that the *nmlR-adhC-estD* operon does not appear to play a role in formaldehyde detoxification in *N. gonorrhoeae* (see Organization and Functional Regulation of Genes in the Glutathione-Dependent Pathways). The gonococcal *adhC* gene is inactive as a consequence of a frameshift mutation (Potter et al., 2007). A link between *nmlR*, *adhC*, or *estD* to formaldehyde detoxification in *N. gonorrhoeae* has not been reported (Kidd et al., 2005; Potter et al., 2007, 2009). Instead, the NmlR regulon in this bacterium has been linked to the response to general thiol/disulfide stress. Mutants of the *estD* gene were sensitive to killing by agents that induce nitrosative stress, such as nitrite and GS-NO (Potter et al., 2009). An *nmlR* mutant also displayed a growth defect in the presence generators of oxidative stress such as cumene hydroperoxide and the thiol oxidant diamide (Kidd et al., 2005).

It is tempting to hypothesize that the apparent divergence of function between two closely related pathogens may relate to their different infection niches. While *N. gonorrhoeae* colonizes the mucosal surfaces of the genitourinary tract, *N. meningitidis* and *H. influenzae* both colonize the nasopharynx, and they are able to cause invasive disease including meningitis and septicemia. The loss of *adhC* in *N. gonorrhoeae* and the conservation of a fully functional *nmlR-adhC-estD* locus in the meningococcus and *H. influenzae* may be an example of a positive selective pressure for this locus during bacteria-host interaction within the nasopharynx. This selection pressure may arise as an indirect consequence of conditions that predispose the invading pathogen to the production of endogenous formaldehyde. Additionally, the potential existence of formaldehyde in the host tissue at the site of infection must also be considered.

### Potential Sources of Formaldehyde

#### Bacterial-Derived Sources

**FIGURE 7:**
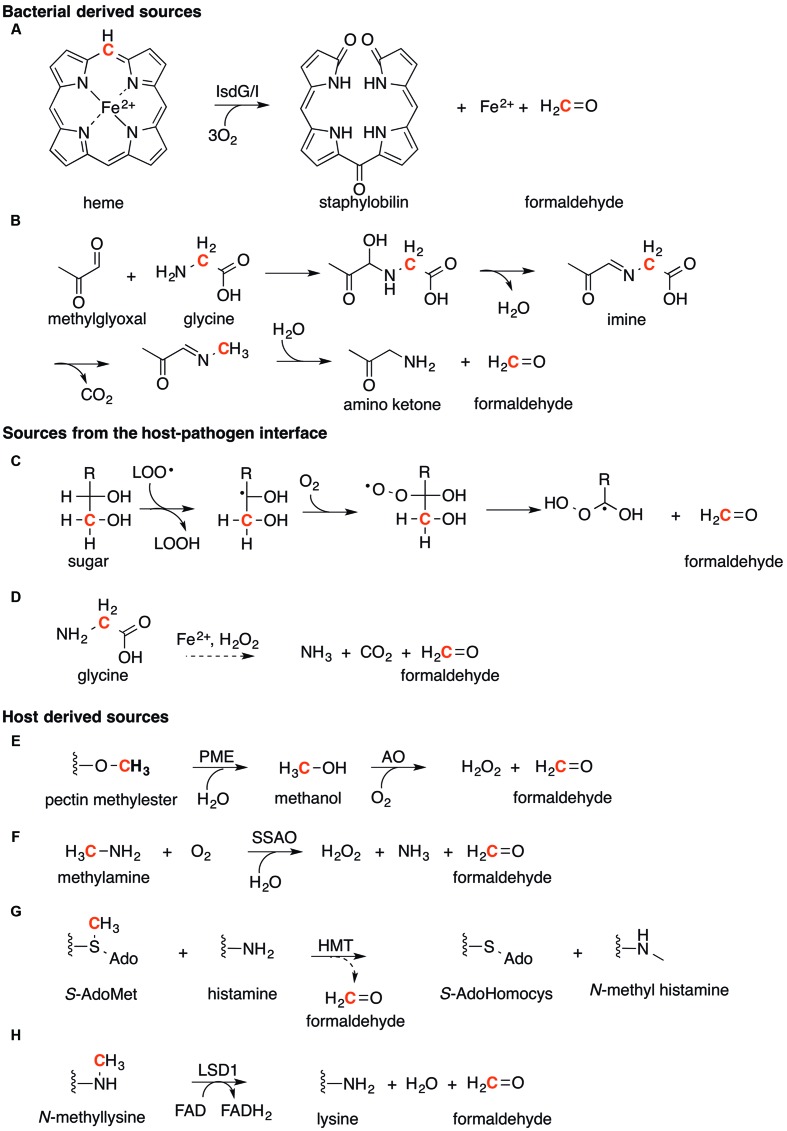
**Sources of formaldehyde.** The generation of formaldehyde can be organized into three main categories. (1) Bacterial derived sources. **(A)** The degradation of heme (substituents omitted for clarity) by IsdG/I, and **(B)** Strecker degradation of glycine with methylglyoxal. (2). Sources from the host-pathogen interface. **(C)** The lipid peroxidation of sugar molecules and **(D)** degradation of glycine by Fenton chemistry. (3) Host derived sources. **(E)** Formaldehyde formed from the oxidation of methanol derived from pectin in fruit. **(F)** Oxidative deamination of methylamine by semicarbazide amine oxidase (SSAO). **(G)** The methyl transfer of histamine by histamine-*N*-methyltransferase (HMT). **(H)** The demethylation of histones by lysine specific demethylase (LSD1). For all reactions, the carbon atom which formaldehyde is generated from is highlighted in Red.

Methylglyoxal, a byproduct of glycolysis, represents a major source of endogenous formaldehyde in bacteria (Schonberg and Moubacher, 1952; Thornalley, 1993; Okado-Matsumoto and Fridovich, 2000). This diketone is produced from the degradation of two triose sugar phosphates, namely glyceraldehyde-3-phosphate and dihydroxyacetone-phosphate (Thornalley, 1993). Methylglyoxal is also formed by the enolization and oxidation of glyceraldehyde, a short-chain sugar of the pentose-phosphate cycle (Okado-Matsumoto and Fridovich, 2000). Generation of formaldehyde from methylglyoxal occurs during Strecker degradation of glycine (**Figure 7A**; Schonberg and Moubacher, 1952). Nucleophilic addition of the amino terminus in glycine to the terminal carbonyl in methylglyoxal reaction creates an imine intermediate, which is subsequently hydrolyzed to generate formaldehyde as the final product.

Production of formaldehyde from methylglyoxal may explain the growth defect of the *adhC* mutant strain of *H. influenzae* under high oxygen tension and in the presence of glucose as the sole carbon source (Kidd et al., 2012). *In silico* analysis of *H. influenzae* has suggested that carbon utilization occurs primarily *via* the pentose phosphate pathway under these conditions (Edwards and Palsson, 1999), leading to the production of methylglyoxal and presumably also formaldehyde.

In the pathogen *S. aureus*, formaldehyde is generated as a byproduct of the degradation of heme during iron acquisition. This process is catalyzed by two heme oxygenases IsdG and IsdI (EC 1.14.99.3, **Figure 7B**; Reniere et al., 2010; Matsui et al., 2013). This process is unique to certain Gram-positive bacteria, including *B. anthracis, S. epidermidis*, and *Listeria monocytogenes* (Skaar et al., 2004), and is distinct from the pathway for heme degradation in Gram-negative bacteria including *H. influenzae*, which generates carbon dioxide in place of formaldehyde (Tenhunen et al., 1969). Analysis of the *S. aureus* genome identified the presence of a complete RuMP pathway for formaldehyde detoxification. It is likely no coincidence that the most abundant source of heme in the human body is in hemoglobin contained in erythrocytes found in blood, the same environment that *S. aureus* can invade and cause disease. Their acquisition of iron from heme in blood would generate increasing amounts of formaldehyde, necessitating for the RuMP based detoxification system.

#### Formaldehyde Generators at the Host–Pathogen Interface

During inflammation, the generation of reactive oxygen species, including the superoxide anion (O_2_^-•^) and hydrogen peroxide (H_2_O_2_), during respiratory burst by macrophages and neutrophils can also produce formaldehyde as a toxic end product (**Figure 7C**). Superoxide and hydrogen peroxide have been demonstrated to damage bacterial iron-sulfur (Fe-S) clusters (Flint et al., 1993; Jang and Imlay, 2007) and mononuclear Fe enzymes (Anjem and Imlay, 2012; Gu and Imlay, 2013), causing the release of Fe as free or bioavailable ions. This bioavailable Fe may catalyze Fenton-like reactions with excess hydrogen peroxide to generate hydroxyl radicals (^•^OH). These radicals in turn may lead to the formation of lipid peroxyl radicals (LOO^•^), which can react with sugars (**Figure 7C**) such as glyceraldehyde in a process that has been shown to produce the toxic aldehydes malondialdehyde and formaldehyde (Cordis et al., 1994; Maboudou et al., 2002). In this process, (**Figure 7C**) initial attack of the lipid peroxide radical with a sugar molecule, followed by reaction with molecular oxygen forms a sugar peroxyl radical. Further rearrangement of this radical occurs to release formaldehyde (Thornalley et al., 1984; Spiteller, 2008). In addition Fenton-catalyzed degradation of L-glycine has been shown to generate formaldehyde (**Figure 7D**) although the precise mechanism is still unknown (Dakin, 1906).

#### Host-Derived Sources

The concentration of formaldehyde in healthy human blood has been measured at 0.1 mM (Heck et al., 1985). This aldehyde is produced by multiple metabolic processes in human and mammalian cells, as described below:

##### Oxidation of methanol by alcohol oxidases

Ingestion of fruits such as apples has been shown to lead to a 10-fold increase in methanol concentration in human breath (Lindinger et al., 1997). This methanol is produced by the hydrolysis of methyl esters in pectins as catalyzed by pectin methylesterases from gut bacteria (PME, EC 3.1.1.11, **Figure 7E**; Siragusa et al., 1988). Methanol is in turn oxidized by human alcohol oxidases (EC 1.1.3.13) to generate formaldehyde as the end product (Mani et al., 1970).

##### Oxidative deamination of primary amines by amine oxidases

Deamination of methylamine by semicarbazide-sensitive amine oxidase (SSAO, EC 1.4.3.6, **Figure 7F**) produces formaldehyde and hydrogen peroxide (Yu and Zuo, 1996). Methylamine itself is produced from deamination of adrenaline, an important hormone and neurotransmitter; sarcosine, a product of glycine biosynthesis; or creatinine, a product of muscle breakdown. This primary amine has been detected in the blood, urine, and brain tissue (Asatoor and Kerr, 1961; Zeisel et al., 1983; Yu et al., 2003). Similarly, the enzyme SSAO is found primarily in blood vessels, although it has also been detected in the meninges and the microvessels of the brain (Zuo and Yu, 1994).

##### Transfer of methyl groups by methyltransferases

Methylation of the neurotransmitter histamine using *S*-adenosylmethionine as the methyl donor is catalyzed by histamine-*N*-methyltransferase (HMT, EC 2.1.1.43, **Figure 7G**). *N*-methylhistamine is generated as the final product but formaldehyde is produced as an intermediate during catalysis (Meller et al., 1974; Huszti and Tyihak, 1986). Significantly, like SSAO, HMT activity has been detected in adult human brain (Nowak and Zelazowska, 1987). Formaldehyde is also generated as an end product of the demethylation of histones by histone lysine specific demethylase 1 (LSD1, EC 1.14.11.27, **Figure 7H**), a nuclear homolog of amine oxidases (Shi et al., 2004). This reaction is likely ubiquitous in all human tissues, as it is crucial for the DNA packing in the nucleus, DNA repair, general stress response, and aging (Greer and Shi, 2012),

## Summary and Outlook

**FIGURE 8:**
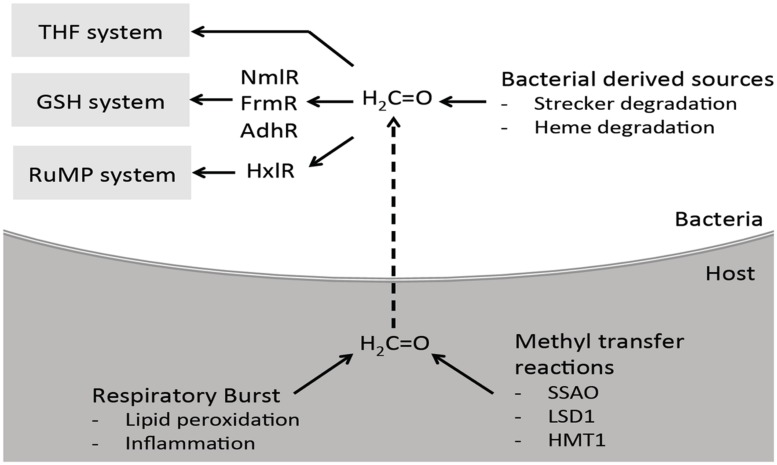
**Proposed interaction and clearance of formaldehyde by bacteria at the host-pathogen interface.** During infection, bacteria may encounter formaldehyde produced endogenously by themselves, and also by the host cells they are infecting. Bacterial endogenously derived sources include heme degradation and Strecker degradation of glycine. The immune system can indirectly release formaldehyde as a consequence of their respiratory burst leading to inflammation and lipid peroxidation. Methyl transfer reactions by host enzymes also contribute to the overall formaldehyde pool. To combat the formaldehyde, bacteria are able to sense and detoxify formaldehyde using the GSH and THF dependent or RuMP systems.

It is clear from this review that the ability to sense and detoxify formaldehyde is not limited to environmental organisms that use methane and methanol as a carbon source. It is likely significant that formaldehyde detoxification pathways are also present in host-adapted bacterial pathogens that were not previously expected to encounter formaldehyde during their physiology. However, it is now recognized that there is a variety of formaldehyde generators at the host-pathogen interface (**Figure 8**). This can be a consequence of the metabolism and growth of the pathogenic bacteria, the host innate immune response and respiratory burst, or the natural metabolic reactions of the infection sites.

Some of these sources of formaldehyde are concentrated in the blood, brain, and surrounding tissues, placing them within the same approximate niche with *N. meningitidis* and *H. influenzae* during the later stages in their infection cycle. The function of NmlR, AdhC, and EstD in these pathogens may contribute to systemic dissemination from the nasopharynx into the blood stream and, ultimately, the brain, which is often associated with invasive disease. We have also shown evidence of the possible transfer of the formaldehyde sensitive *nmlR* regulon from pathogenic *Neisseria* to *Haemophilus* species. Whether the presence of formaldehyde within the nasopharynx directly influenced this transfer is still unknown.

In addition, *E. coli*, including pathogenic strains, *P. aeruginosa*, and *K. pneumoniae*, possess the FrmRAB regulon, while the RuMP pathway is present in *L. monocytogenes* and *S. aureus*, and co-factor independent formaldehyde dehygrogenases have been identified in the opportunistic pathogens *P. aeruginosa* and *P. putida* The formaldehyde detoxifications systems found in these medically significant pathogens are very likely required during pathogenesis to remove the endogenous and exogenously produced formaldehyde, however, this contribution still remains to be tested empirically.

The precise mechanism of how they sense formaldehyde requires further investigation, as does measurement of the intracellular formaldehyde in bacterial pathogens and at the host–pathogen interface. Additional further testing of mutants in these detoxification systems in host infection models and global transcriptome analysis would be useful to determine how great of an extent they are required for overall survival. Continued investigation into the role of formaldehyde during host-pathogen interactions will no doubt be useful to further understanding the already complex field of bacterial pathogenesis.

## Author Contributions

AM conceived the manuscript. NC performed the literature review. NC, KD, FV and AM co-wrote the manuscript. NC, KD and AM performed the final review and editing.

## Conflict of Interest Statement

The authors declare that the research was conducted in the absence of any commercial or financial relationships that could be construed as a potential conflict of interest.
